# eFAST for the diagnosis of a perioperative complication during percutaneous nephrolithotomy

**DOI:** 10.1186/s13089-018-0088-1

**Published:** 2018-04-03

**Authors:** Achyut Sharma, Prajjwal Bhattarai, Apurb Sharma

**Affiliations:** Nepal Mediciti Hospital, Sainbu, Bhaisepati, Lalitpur, Nepal

**Keywords:** eFAST, Percutaneous nephrolithotomy, Fluid extravasation, Perioperative ultrasound

## Abstract

A 29-year-old patient with normal preanesthetic evaluation was planned for percutaneous nephrolithotomy (PCNL) for right nephrolithiasis with right pyelolithiasis. Surgery was performed under general anesthesia with endotracheal intubation with muscle relaxation. At the conclusion of surgery, when the patient was turned over to supine position, tense abdomen was noted. Immediately extended focused assessment with sonography in trauma (eFAST) was done in which both right and left quadrants of abdomen including pericardial and suprapubic region, right and left thoracic, and both lung basis were examined. Fluid collection was seen in Morison’s pouch which was drained by the urologist under real-time ultrasonography guidance by anesthesiologist. Distension of abdomen subsequently subsided and patient had normal vitals. Trachea was extubated and patient shifted to post-operative ward. eFAST and FAST scans are routine procedures in the rapid assessment of trauma victims in emergency settings. The fluid extravasation during a routine PCNL procedure may lead to abdominal compartment syndrome. This case demonstrated that use of eFAST rapidly detected abdominal collection and ruled out life-threatening conditions such as hemothorax and pneumothorax and prevented abdominal compartment syndrome. Our case is only an example that potentially lethal conditions like these may be encountered in the perioperative setting and the knowledge of eFAST scan may be of great help.

**Electronic supplementary material:**

The online version of this article (10.1186/s13089-018-0088-1) contains supplementary material, which is available to authorized users.

## Background

Complications such as abdominal compartment syndrome are rare but potentially life-threatening conditions [1] that occur during percutaneous nephrolithotomy (PCNL) and require urgent diagnosis and treatment to prevent morbidity and mortality. The use of ultrasound and rapid assessment of intraabdominal fluid collection by the use of eFAST (extended focused assessment with sonography in trauma) in the perioperative settings help achieve this feat. We report a case where the use of eFAST helped diagnose and promptly treat one such serious complication of PCNL within the operation theater.

## Case presentation

A 29-year-old gentleman was admitted to our center with the provisional diagnosis of right nephrolithiasis with right pyelolithiasis with right gross hydronephrosis. He was planned for right-sided PCNL. Preoperative anesthetic evaluation did not reveal abnormal findings except for high total leucocyte count during routine urine examination which was normalized after 5-day course of inj. amikacin. There was no significant medical, surgical, family, and psychosocial history.

In the operation theater, anesthesia was induced with propofol and trachea was intubated with size 7.5 mm ID flexometallic endotracheal cuffed tube using vecuronium 7 mg as muscle relaxant. Maintenance was done by isoflurane, oxygen, and intermittent vecuronium top-ups. The initial intrathoracic pressure was 19 cm of H_2_O. Patient was kept on volume control mode with initial tidal volume of 8 ml/kg. Over the period of surgery of 2 h, a gradual increase in intrathoracic pressure from 19 cm of H_2_O to 30 cm of H_2_O was noted. It was ascertained intraoperatively that there was no pneumothorax by auscultation of chest and confirming the presence of breath sounds antero-posteriorly. Other potential causes of rise in intrathoracic pressure such as endotracheal tube displacement, kinking, and obstruction due to secretions were ruled out immediately. The large numbers of stones and their fragments required further surgical time and thus the initial decrease of tidal volume to 6 ml/kg was done to decrease the intrathoracic pressure. The rest of the intraoperative period was uneventful.

When the patient was turned supine, it was noted that patient had a tense abdomen. Initial impression led us to believe that there was intraabdominal extravasation of fluid. Immediately eFAST was done in which the left and right thoracic views were done to exclude hemothorax and pneumothorax. Both right and left quadrants of the abdomen were scanned along with the pericardial and suprapubic scan. It was noted that there was significant fluid collection in the Morison’s pouch. Drainage of fluid was then accomplished in real-time ultrasound guidance. Subsequently, the distension of abdomen decreased. After the return of adequate muscle strength, spontaneous breathing, and normalization of intrathoracic pressure, trachea was extubated and patient was shifted to post-operative ward.

In the post-operative ward, patient was managed conservatively with analgesics, antibiotics, fluids, and diuretics. Vitals including abdominal girth were measured continuously until the patient was shifted to ward. A repeat ultrasonogram done by radiologist 2 days later showed fluid collection in Morrison’s pouch, left upper abdomen, and bilateral iliac fossa but the collection was smaller in volume. Patient subsequently improved with conservative management and was discharged. Patient’s condition had normalized in the follow-up visit a week later.

## Discussion

With the ever-growing complexity and extensiveness in surgical procedures, it is not uncommon to encounter unique perioperative complications. Ours is an exemplary case describing the chances of occurrence of grave consequences in a seemingly simple and minimally invasive procedure. No matter how trivial the problem is, how ordinary the operative procedure is, every case is a challenge to anesthesiologist. Knowing how to operate and interpret results of ultrasound which have become a commonplace in operation theaters and ICU these days helps save lives.

It is undisputed that ultrasound is one of the most sensitive, effective and reliable tools to assess any trauma victim; all thanks to the eFAST and FAST scan in trauma series [1]. While classically in FAST scan included four areas for fluid collection, namely perihepatic space (called the Morison’s pouch), perisplenic space, pericardium and the pelvis, the eFAST allowed the examination of both lungs by adding bilateral anterior thoracic sonography to the FAST exam. eFAST is not only quick and easy to perform, it is as specific as chest X-rays but more sensitive than the latter in terms of detection of occult pneumothoraces after trauma [2]. To perform the eFAST, the patient is placed in supine position and with the use of a convex transducer 3.5–5.0 MHz several regions are scanned. The subxiphoid transverse view assesses for pericardial effusion and injuries to left lobe of liver; the longitudinal view of the right upper quadrant assesses for right liver injury, right kidney injury, and Morison’s pouch; the longitudinal view of left upper quadrant is assessed to see for splenic injuries and left kidney injury; the transverse and longitudinal views of the suprapubic region are used to assess bladder and pouch of Douglas; finally, the right and left thoracic views, and both lung basis are obtained to assess for pneumothorax and hemothorax. Although the various organ and cavity scans may seem overwhelming when it comes to the necessity of a quick diagnosis of life-threatening complications, if the technique can be protocolized as to how and which to be scanned, the times can be reduced greatly. In addition, with the frequent involvement in trainings and reinforcement program, any physician can keep up the skill and perform such scan relatively quickly. The time we spent in eFAST scan was around 2 min.

However, in the developing countries, the perioperative use of ultrasound has, until now, been limited to USG-guided regional anesthesia [3] and vascular access [4]. There are reports of its use in other perioperative settings [5]. But the relatively easy access to USG these days, its non-invasiveness, and the availability of personnel such as anesthesiologists who are equipped with knowledge and expertise required to operate and interpret such equipment extend the utilities of such machines. No wonder, it becomes imperative for any anesthesiologist involved in the perioperative care to be acquainted with the ultrasonographic examination as it aids us in rapid assessment of various intraabdominal and intrathoracic pathologies. Although the diagnosis by clinical assessment cannot be superseded by any forms of diagnostic tests, it, however, can be complemented by the use of aids such as ultrasound and, therefore, must be used more frequently.

Extravasation of irrigation fluid during PCNL is not an uncommon finding. Some case reports of massive extravasation of irrigation fluid leading to abdominal compartment syndrome have also been reported [6, 7]. The gradually increasing airway pressures might have suggested pneumothorax or abdominal compartment syndrome. However, it is often difficult to distinguish between various causes of increased intrathoracic pressure such as displacement, blockage of tube due to secretions, and bronchospasm; especially when the patient is in prone position. Proper anesthetic technique, intensive monitoring for airway pressures and immediate diagnosis of fluid extravasation help prevent such unfortunate incidents of abdominal compartment syndrome. The use of ultrasound is helpful in preventing and detecting most of these complications. In our case, the most obvious sign of abdominal distension to detect extravasation was not helpful as the patient was in prone position which made the evaluation difficult initially.

The easy accessibility to the ultrasound and our prompt intervention helped prevent a condition from worsening further as we could drain the abdominal fluid relatively easily under the USG guidance. The ominous signs of pneumothorax and hemothorax were also ruled out expeditiously with the aid of ultrasound. If this was not the case, then we would have had to transfer the patient to radiology suite which would have consumed our valuable time and thus leading to formidable consequences. Certainly, the use of ultrasound in the perioperative settings cannot be understated (Additional files 1, 2).

## Conclusion

Perioperative ultrasound in the form of eFAST scan can be readily used in detecting complications such as abdominal extravasation of fluid in surgeries such as percutaneous nephrolithotomy. This use of ultrasound within the operation theater certainly saves time and life.

## Additional files

**Additional file 1.** The USG clip demonstrating the negotiation of drain tube.

**Additional file 2.** The drain tube can be seen within the Morison's pouch.

## Abbreviations

eFAST: extended focused assessment with sonography in trauma
FAST: focused assessment with sonography in trauma
H_2_O: water
ICU: intensive care unit
MHz: megahertz
PCNL: percutaneous nephrolithotomy
USG: ultrasonography

## References

[1] Hamada SR, Delhaye N, Kerever S, Harrois A, Duranteau J (2016). Integrating eFAST in the initial management of stable trauma patients: the end of plain film radiography. Ann Intensive Care.

[2] Kirkpatrick AW, Sirois M, Laupland KB, Liu D, Rowan K, Ball CG (2004). Hand-held thoracic sonography for detecting post-traumatic pneumothoraces: the extended focused assessment with sonography for trauma (EFAST). J Trauma.

[3] Bhattarai PR, Rayamajhi AJ, Yadav RK, Paudel SC, Pangeni A, Byanjankar B (2017). Comparison of ultrasound-guided abdominal nerve blocks and subarachnoid block as an anaesthetic technique for appendectomy: a retrospective study. J Soc Anesthesiol Nepal.

[4] Parajuli S, Sharma A (2017). Ultrasound-guided internal jugular catheterisation in paediatric cardiac surgical patients: a prospective observational study. Egypt J Cardiothorac Anesth.

[5] Jung A, Civil R, Hospital S, Raj P, Civil B, Hospital S (2016). Role of lung scan for detection of pneumothorax in operation theatre. Birat J Health Sci.

[6] Tao J, Sheng L, Zhang H, Chen R, Sun Z, Qian W (2016). Urology case reports acute abdominal compartment syndrome as a complication of percutaneous nephrolithotomy: two cases reports and literature review. Urol Case Rep.

[7] Etemadian M, Shadpour P, Haghighi R, Mokhtari MR, Maghsoudi R (2012). A rare, but life-threatening complication of percutaneous nephrolithotomy. Urol J.

