# Supplementary figures and images for: eFAST for the diagnosis of a perioperative complication during percutaneous nephrolithotomy
Source: Crit Ultrasound J. 2018 Apr 3;10:7. doi: 10.1186/s13089-018-0088-1 (PMC5882478; doi:10.1186/s13089-018-0088-1)

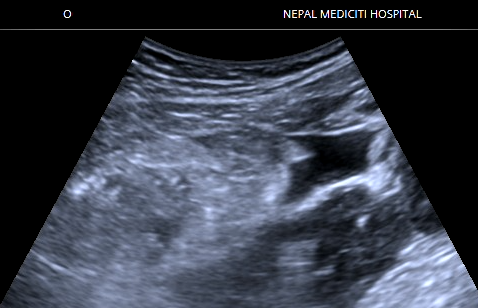

Supplement: Supplementary file 2 — Additional file 2. The drain tube can be seen within the Morison's pouch. [file 13089_2018_88_MOESM2_ESM.png]
